# Supplementary material for: Contribution of ADD3 and the HLA Genes to Biliary Atresia Risk in Chinese
Source: Int J Mol Sci. 2023 Sep 29;24(19):14719. doi: 10.3390/ijms241914719 (PMC10572496; doi:10.3390/ijms241914719)
Supplement: Supplementary file 1 [file ijms-24-14719-s001.zip › ijms-2598824-Supplementary Figures S1-S7.pdf]

Supplementary Figures

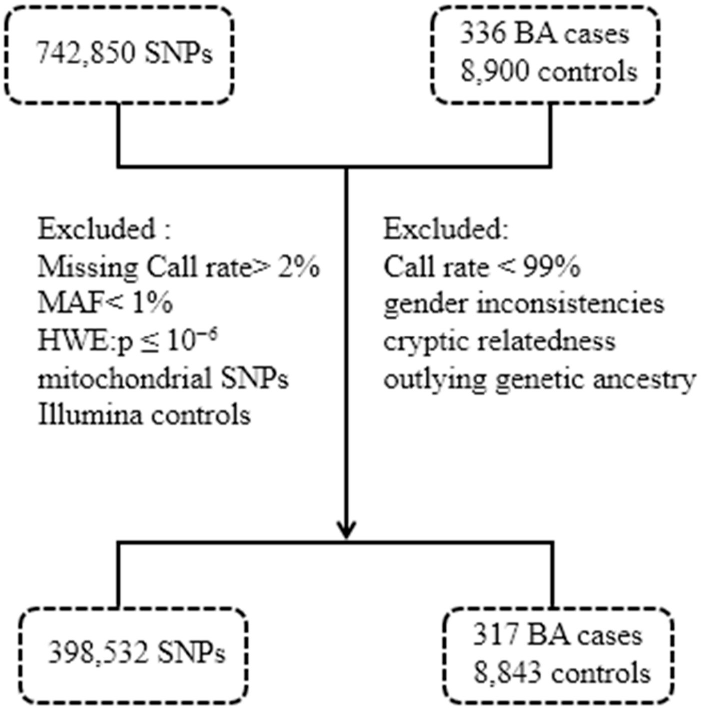

Figure S1. The flowchart for quality filtering in GWAS data. Quality filtering was performed on SNPs and samples before analysis to ensure robust association tests.

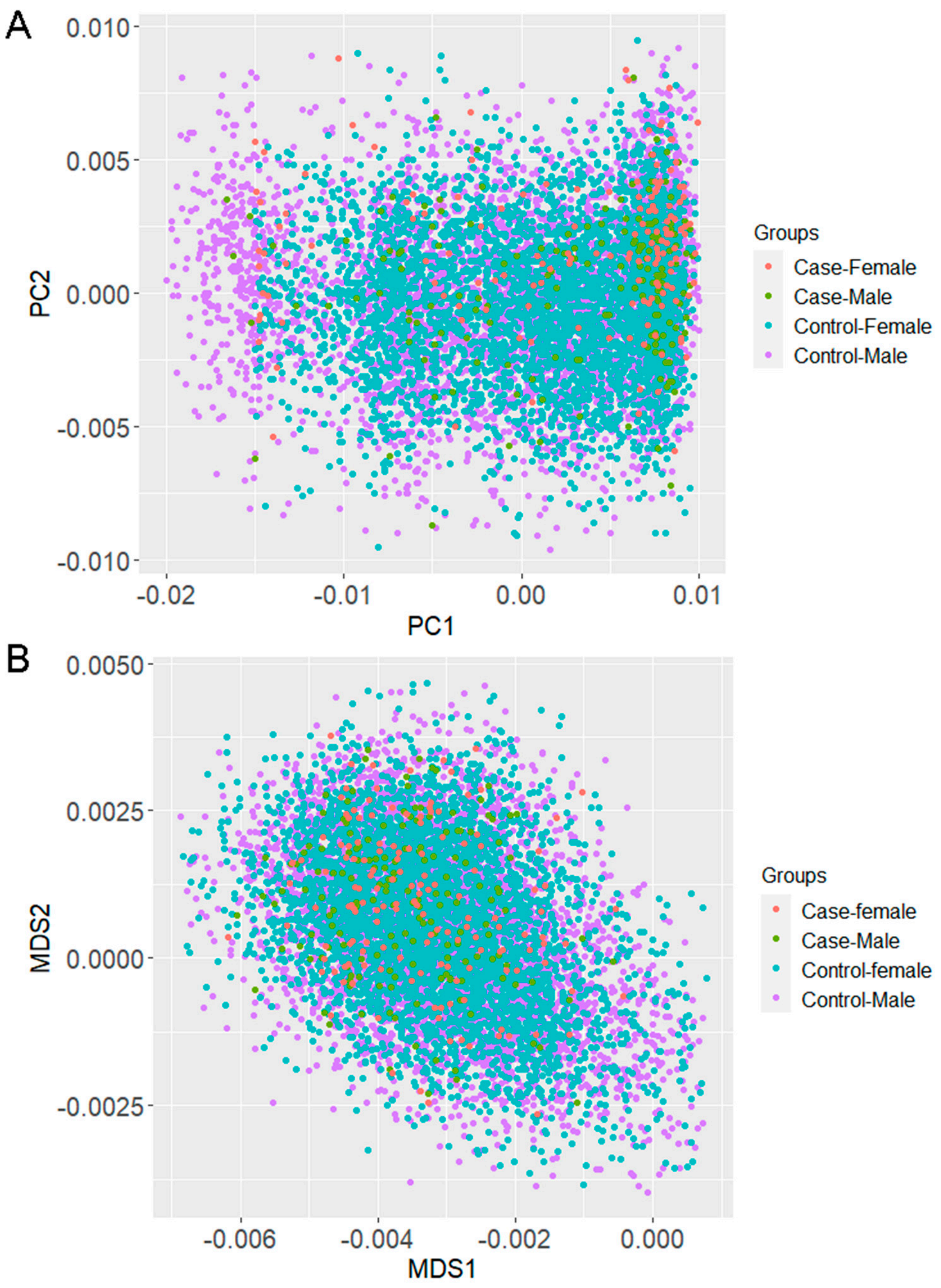

Figure S2. Plots of principal component analysis (PCA) and multidimensional scaling (MDS) analysis of GWAS samples. (**A**) Principal components of 317 BA cases and 8,843 controls were plotted using the first two eigenvectors produced by the EIGENSOFT software. (**B**) The first and second dimension values of 317 BA cases and 8,843 controls were produced by PLINK. The identity-by-descent (IBD) pairwise distances among all the samples were used to construct dimensions and MDS analysis (http://pngu.mgh.harvard.edu/purcell/plink/). No significant structures were observed in the current cohort.

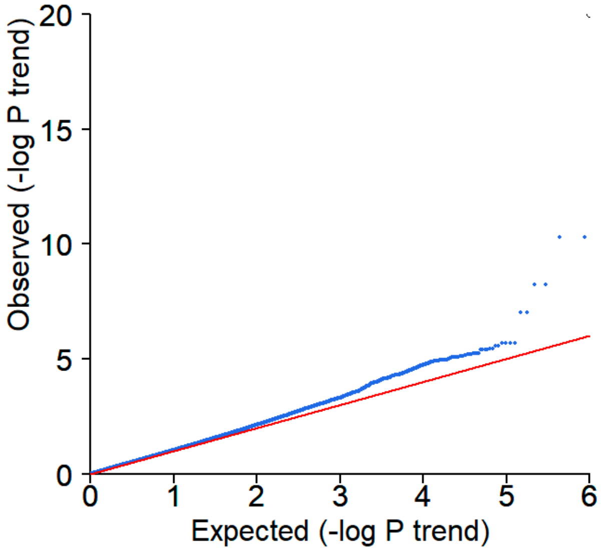

Figure S3. Quantile-quantile (Q-Q) plot of GWAS results for BA risk

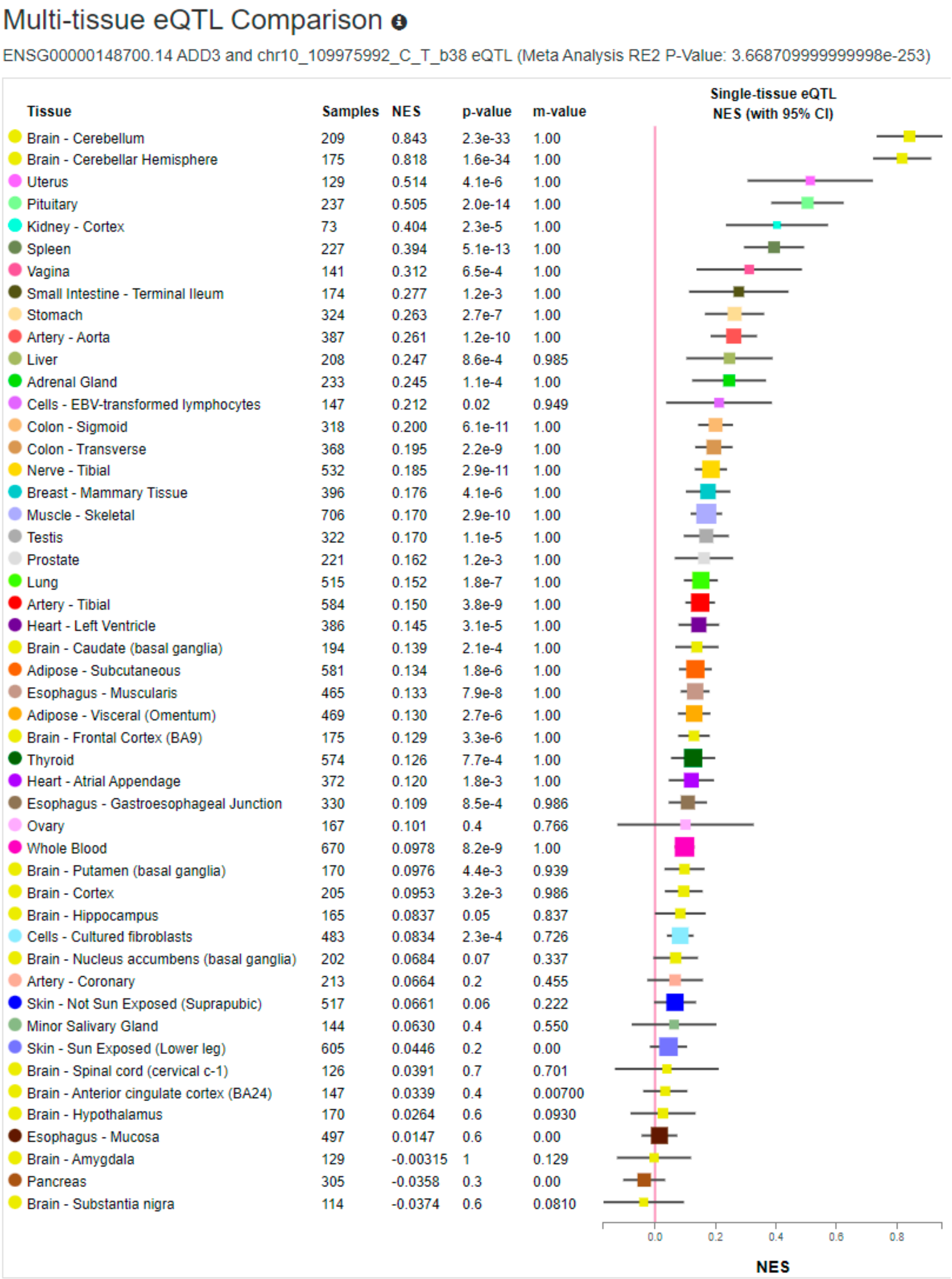

Tissue	Samples	NES	p-value	m-value
Brain - Cerebellum	209	0.843	2.3e-33	1.00
Brain - Cerebellar Hemisphere	175	0.818	1.6e-34	1.00
Uterus	129	0.514	4.1e-6	1.00
Pituitary	237	0.505	2.0e-14	1.00
Kidney - Cortex	73	0.404	2.3e-5	1.00
Spleen	227	0.394	5.1e-13	1.00
Vagina	141	0.312	6.5e-4	1.00
Small Intestine - Terminal Ileum	174	0.277	1.2e-3	1.00
Stomach	324	0.263	2.7e-7	1.00
Artery - Aorta	387	0.261	1.2e-10	1.00
Liver	208	0.247	8.6e-4	0.985
Adrenal Gland	233	0.245	1.1e-4	1.00
Cells - EBV-transformed lymphocytes	147	0.212	0.02	0.949
Colon - Sigmoid	318	0.200	6.1e-11	1.00
Colon - Transverse	368	0.195	2.2e-9	1.00
Nerve - Tibial	532	0.185	2.9e-11	1.00
Breast - Mammary Tissue	396	0.176	4.1e-6	1.00
Muscle - Skeletal	706	0.170	2.9e-10	1.00
Testis	322	0.170	1.1e-5	1.00
Prostate	221	0.162	1.2e-3	1.00
Lung	515	0.152	1.8e-7	1.00
Artery - Tibial	584	0.150	3.8e-9	1.00
Heart - Left Ventricle	386	0.145	3.1e-5	1.00
Brain - Caudate (basal ganglia)	194	0.139	2.1e-4	1.00
Adipose - Subcutaneous	581	0.134	1.8e-6	1.00
Esophagus - Muscularis	465	0.133	7.9e-8	1.00
Adipose - Visceral (Omentum)	469	0.130	2.7e-6	1.00
Brain - Frontal Cortex (BA9)	175	0.129	3.3e-6	1.00
Thyroid	574	0.126	7.7e-4	1.00
Heart - Atrial Appendage	372	0.120	1.8e-3	1.00
Esophagus - Gastroesophageal Junction	330	0.109	8.5e-4	0.986
Ovary	167	0.101	0.4	0.766
Whole Blood	670	0.0978	8.2e-9	1.00
Brain - Putamen (basal ganglia)	170	0.0976	4.4e-3	0.939
Brain - Cortex	205	0.0953	3.2e-3	0.986
Brain - Hippocampus	165	0.0837	0.05	0.837
Cells - Cultured fibroblasts	483	0.0834	2.3e-4	0.726
Brain - Nucleus accumbens (basal ganglia)	202	0.0684	0.07	0.337
Artery - Coronary	213	0.0664	0.2	0.455
Skin - Not Sun Exposed (Suprapubic)	517	0.0661	0.06	0.222
Minor Salivary Gland	144	0.0630	0.4	0.550
Skin - Sun Exposed (Lower leg)	605	0.0446	0.2	0.00
Brain - Spinal cord (cervical c-1)	126	0.0391	0.7	0.701
Brain - Anterior cingulate cortex (BA24)	147	0.0339	0.4	0.00700
Brain - Hypothalamus	170	0.0264	0.6	0.0930
Esophagus - Mucosa	497	0.0147	0.6	0.00
Brain - Amygdala	129	-0.00315	1	0.129
Pancreas	305	-0.0358	0.3	0.00
Brain - Substantia nigra	114	-0.0374	0.6	0.0810

Figure S4. eQTL plot for rs17095355 genotypes related with *ADD3* expression. The plot was based on genotype and transcriptome data from the GTEx Analysis Release V8 (dbGaP Accession phs000424.v8.p2). Rs17095355 risk allele T is associated with increased level of *ADD3* expression in most of the body tissues. Spleen (n = 227, P_{GTEx} = 5.1×10^{-13}); Whole blood (n = 670, P_{GTEx} = 8.2×10^{-9}). See also in Aging (Albany NY). 2020 Apr 21;12(8):7163-7182.

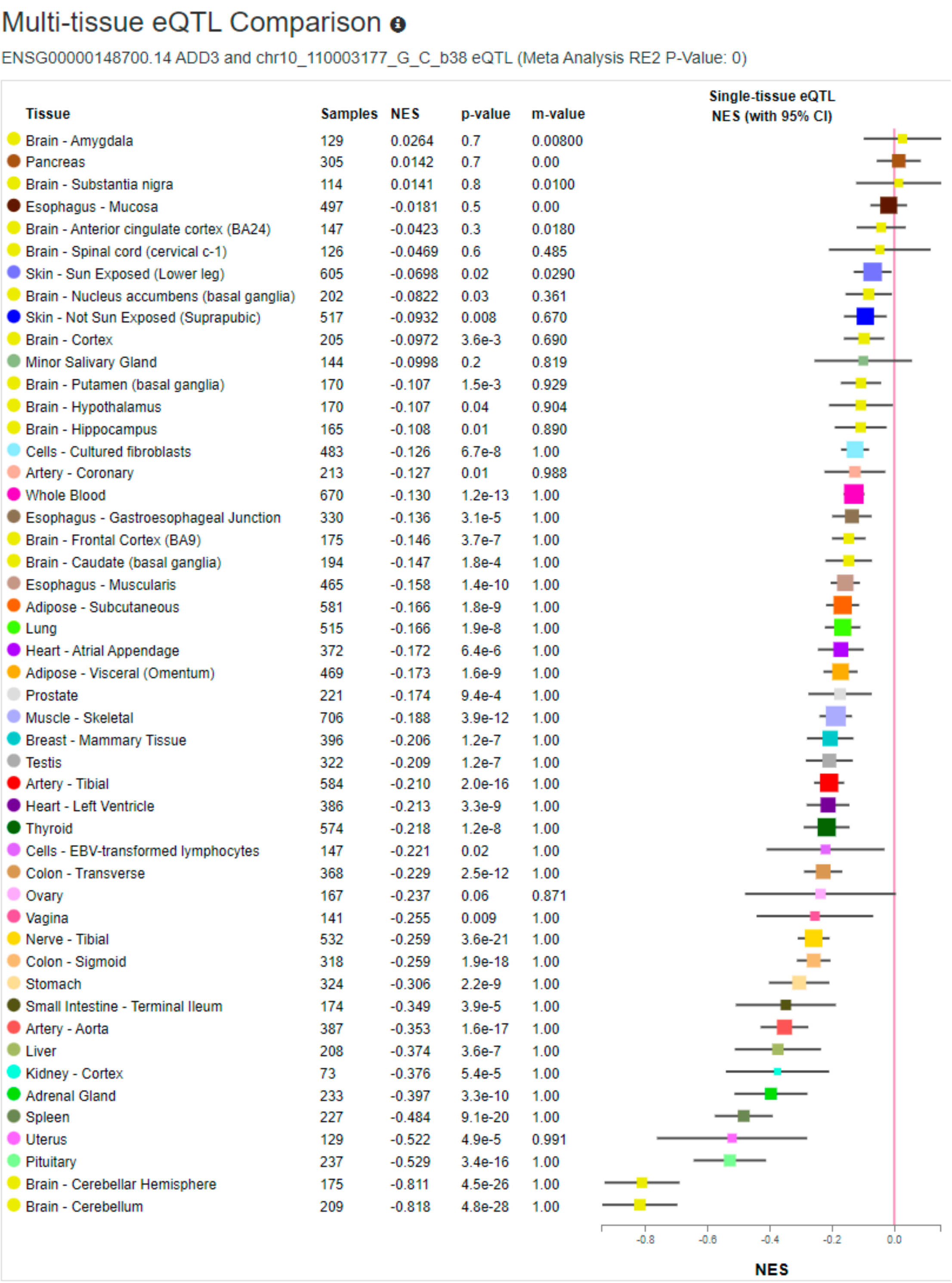

Multi-tissue eQTL Comparison

ENSG00000148700.14 ADD3 and chr10_110003177_G_C_b38 eQTL (Meta Analysis RE2 P-Value: 0)

Tissue	Samples	NES	p-value	m-value
Brain - Amygdala	129	0.0264	0.7	0.00800
Pancreas	305	0.0142	0.7	0.00
Brain - Substantia nigra	114	0.0141	0.8	0.0100
Esophagus - Mucosa	497	-0.0181	0.5	0.00
Brain - Anterior cingulate cortex (BA24)	147	-0.0423	0.3	0.0180
Brain - Spinal cord (cervical c-1)	126	-0.0469	0.6	0.485
Skin - Sun Exposed (Lower leg)	605	-0.0698	0.02	0.0290
Brain - Nucleus accumbens (basal ganglia)	202	-0.0822	0.03	0.361
Skin - Not Sun Exposed (Suprapubic)	517	-0.0932	0.008	0.670
Brain - Cortex	205	-0.0972	3.6e-3	0.690
Minor Salivary Gland	144	-0.0998	0.2	0.819
Brain - Putamen (basal ganglia)	170	-0.107	1.5e-3	0.929
Brain - Hypothalamus	170	-0.107	0.04	0.904
Brain - Hippocampus	165	-0.108	0.01	0.890
Cells - Cultured fibroblasts	483	-0.126	6.7e-8	1.00
Artery - Coronary	213	-0.127	0.01	0.988
Whole Blood	670	-0.130	1.2e-13	1.00
Esophagus - Gastroesophageal Junction	330	-0.136	3.1e-5	1.00
Brain - Frontal Cortex (BA9)	175	-0.146	3.7e-7	1.00
Brain - Caudate (basal ganglia)	194	-0.147	1.8e-4	1.00
Esophagus - Muscularis	465	-0.158	1.4e-10	1.00
Adipose - Subcutaneous	581	-0.166	1.8e-9	1.00
Lung	515	-0.166	1.9e-8	1.00
Heart - Atrial Appendage	372	-0.172	6.4e-6	1.00
Adipose - Visceral (Omentum)	469	-0.173	1.6e-9	1.00
Prostate	221	-0.174	9.4e-4	1.00
Muscle - Skeletal	706	-0.188	3.9e-12	1.00
Breast - Mammary Tissue	396	-0.206	1.2e-7	1.00
Testis	322	-0.209	1.2e-7	1.00
Artery - Tibial	584	-0.210	2.0e-16	1.00
Heart - Left Ventricle	386	-0.213	3.3e-9	1.00
Thyroid	574	-0.218	1.2e-8	1.00
Cells - EBV-transformed lymphocytes	147	-0.221	0.02	1.00
Colon - Transverse	368	-0.229	2.5e-12	1.00
Ovary	167	-0.237	0.06	0.871
Vagina	141	-0.255	0.009	1.00
Nerve - Tibial	532	-0.259	3.6e-21	1.00
Colon - Sigmoid	318	-0.259	1.9e-18	1.00
Stomach	324	-0.306	2.2e-9	1.00
Small Intestine - Terminal Ileum	174	-0.349	3.9e-5	1.00
Artery - Aorta	387	-0.353	1.6e-17	1.00
Liver	208	-0.374	3.6e-7	1.00
Kidney - Cortex	73	-0.376	5.4e-5	1.00
Adrenal Gland	233	-0.397	3.3e-10	1.00
Spleen	227	-0.484	9.1e-20	1.00
Uterus	129	-0.522	4.9e-5	0.991
Pituitary	237	-0.529	3.4e-16	1.00
Brain - Cerebellar Hemisphere	175	-0.811	4.5e-26	1.00
Brain - Cerebellum	209	-0.818	4.8e-28	1.00

Figure S5. eQTL plot for the imputed SNP rs3862007 genotypes related with *ADD3* expression**.** The plot was based on genotype and transcriptome data from the GTEx Analysis Release V8 (dbGaP Accession phs000424.v8.p2). The rs3862007 risk allele T is associated with increased levels of *ADD3* expression in liver (n = 208, $P_{\mathrm{GTEx}} = 3.6 \times 10^{-7}$), spleen (n = 227, $P_{\mathrm{GTEx}} = 9.1 \times 10^{-20}$) and whole blood (n = 670, $P_{\mathrm{GTEx}} = 1.2 \times 10^{-13}$).

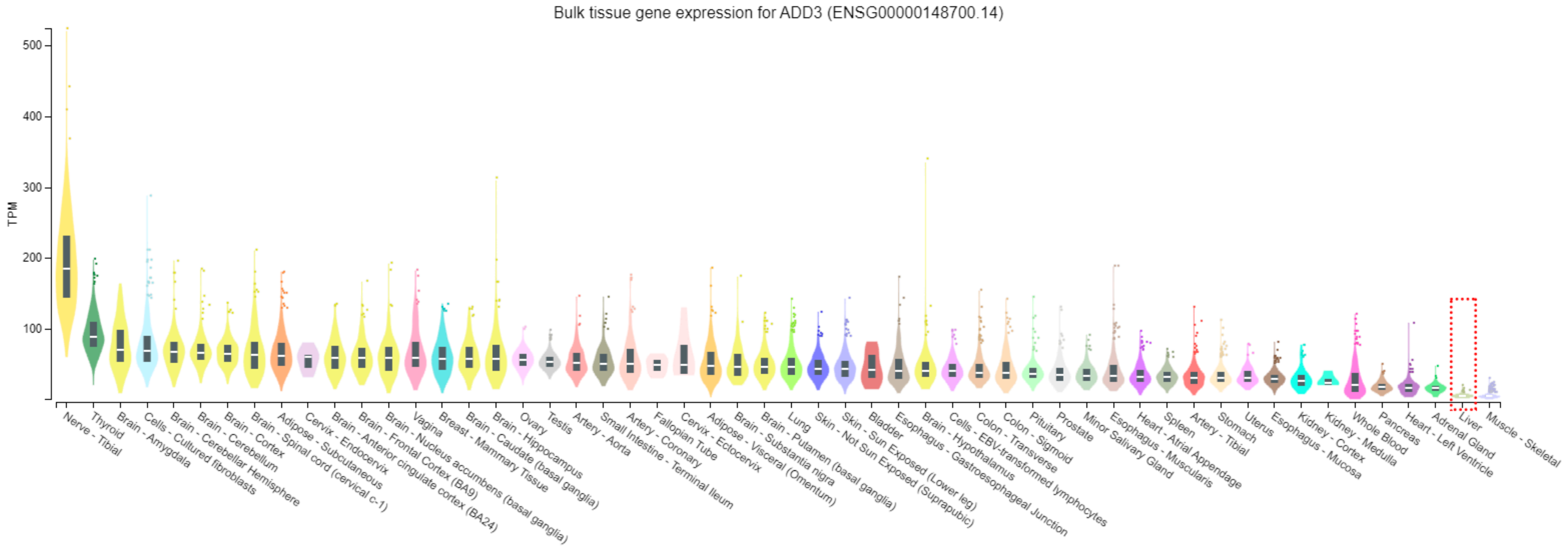

Figure S6. Bulk tissue gene expression for ADD3 (ENSG00000148700.14). Data Source: GTEx Analysis Release V8 (dbGaP Accession phs000424.v8.p2).

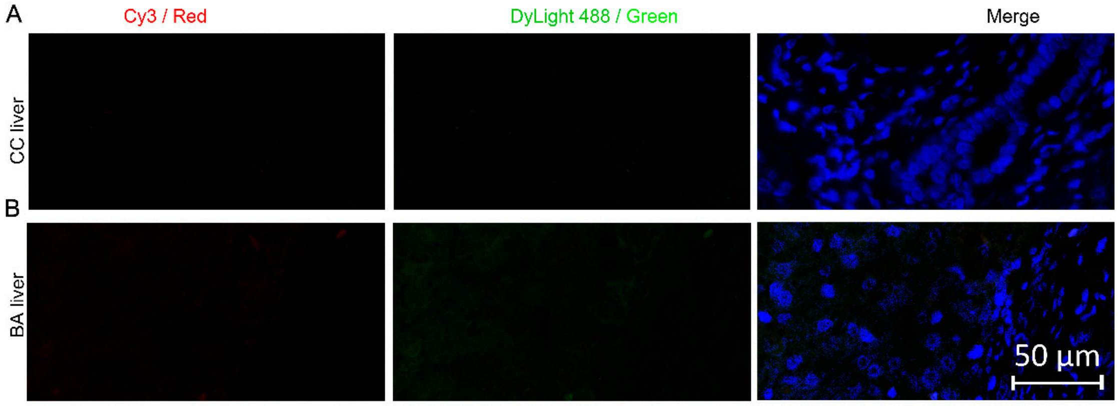

Figure S7. Negative control for immunofluorescence detection of ADD3 in (**A**) choledochal cyst (CC) and (**B**) BA liver sections. Alexa Fluor 488-conjugated rabbit anti-goat IgG (Red) and Cy3-conjugated goat anti-mouse IgG (Green) were used as the secondary antibodies. Blue color: DAPI staining of nuclei. 400× magnification. Scale bar: 50 μm.
